# Colorectal and uterine movement and tension of the inferior hypogastric plexus in cadavers

**DOI:** 10.1186/2045-709X-20-13

**Published:** 2012-04-20

**Authors:** Ian P Johnson

**Affiliations:** 1 Discipline of Anatomy and Pathology, University of Adelaide, Frome Road, Adelaide, SA, 5000, Australia

**Keywords:** Cadaver, Endopelvic fascia, Inferior hypogastric plexus, Somatovisceral

## Abstract

**Background:**

Hypotheses on somatovisceral dysfunction often assume interference by stretch or compression of the nerve supply to visceral structures. The purpose of this study is to examine the potential of pelvic visceral movement to create tension of the loose connective tissue that contains the fine branches of the inferior hypogastric nerve plexus.

**Methods:**

Twenty eight embalmed human cadavers were examined. Pelvic visceral structures were displaced by very gentle 5 N unidirectional tension and the associated movement of the endopelvic fascia containing the inferior hypogastric plexus that this caused was measured.

**Results:**

Most movement of the fascia containing the inferior hypogastric plexus was obtained by pulling the rectosigmoid junction or broad ligament of the uterus. The plexus did not cross any vertebral joints and the fascia containing it did not move on pulling the hypogastric nerve.

**Conclusions:**

Uterine and rectosigmoid displacement produce most movement of the fascia containing the hypogastric nerve plexus, potentially resulting in nerve tension. In the living this might occur as a consequence of menstruation, pregnancy or constipation. This may be relevant to somatovisceral reflex theories of the effects of manual therapy on visceral conditions.

## Background

Beneficial effects have been reported for chiropractic and manual therapy treatment of conditions affecting pelvic viscera such as the bowel, bladder and uterus [1-4], although study design often makes meta-analyses difficult [5-7]. In contrast, very little is known about the mechanisms involved. Neural-based theories are often offered as explanations- and sometimes justifications- for treatment [8-10], but there is little human evidence to support them. This weakens the overall rationale for choosing chiropractic and manual therapy protocols for conditions with underlying visceral aetiologies even if outcome studies suggest a benefit. The aim of this study is to test the possibility that movement of pelvic viscera in humans might put mechanical tension on visceral nerves. This may help define the structural basis of some somatovisceral interactions where adverse mechanical tension of nerves is presumed to be operating [8,9]. One likely candidate here is the inferior hypogastric plexus, since it is in close anatomical proximity to- and innervates- much of the pelvic viscera [11,12]. It is known that nerve stretch can alter nerve function [13,14], and there is evidence that even mild stretch within normal limits can alter nerve activity if this occurs in association with local inflammation [15]. In this regard, the questions of whether the inferior hypogastric plexus is in a position to be stretched at all, and if so by what and by how much, may be relevant to the interpretation of somatovisceral theories that assume its altered activity. Unfortunately standard neurophysiological studies of the inferior hypogastric plexus are made very difficult by its location as it is embedded in fascia on the posterior abdominal wall [16]. Anatomical studies, in contrast, are more feasible and could help guide subsequent functional studies. We previously showed that the position of the inferior hypogastric plexus can be located consistently in the cadaver with the use of surgical landmarks [17]. The present study builds upon these observations determine the extent to which the fascia containing the fine branches of this plexus can be put under tension by the movement of pelvic organs. A preliminary report has been published [18].

## Methods

A total of 28 embalmed human cadavers were examined. Pelvic organs were examined *in-situ* in 4 cadavers after opening the abdomen. In the remaining 24, the pelvis was sectioned in the sagittal plane to reveal the pelvic organs more clearly. Using accurate mid-line hemisection and good preservation of the pelvic viscera as inclusion criteria, 18 (12 male and 6 female) of these sagittally-sectioned cadavers were selected for semi-quantitative analyses. The same 6 female hemipelves and 6 male hemipelves (randomly-selected from the 12 used for semi-quantitative analyses) were used for quantitative measurements of endopelvic fascial movement. All work was undertaken within the scope of the UK Anatomy Act 1983 and Human Tissue Act 2005 [19,20] and had local institutional approval.

The position of the plexus in the endopelvic fascia was taken as two fingers breadth lateral to the third anterior sacral foramina, lying deep to a line drawn from the thirds sacral vertebra, the conventional level of the rectosigmoid junction, and the palpable posterior surface of the pubic symphysis [17]. Taking care to remain superficial to the endopelvic fascia, a note was made of the relations of this fascia and the ease with which displacement of this fascia could be produced by gentle movement of related visceral structures to an extent that was considered to reflect the range possible during life. Any movement that caused the endopelvic fascia to tear was considered abnormal and not considered further here. Even for relatively fixed structures, such as the prostate, approximately 1 cm movement was easily achieved in embalmed cadavers without any macroscopic damage or disruption to the fascia. While there are no studies concerned specifically with the range of movement normally found for human pelvic viscera in the living, it can be estimated by direct observation of the adult colorectal anatomy that approximately 10 cm movement must occur in the rectum and colon to allow the advancement of a rigid probe as is the case for a rigid sigmoidoscope. Similarly, filling of the urinary bladder with a normal anteflexed and anteverted uterus superior to it must on anatomical grounds result in the uterine fundus tracing an arc of at least 10 cm in the adult. This is supported by consecutive MRI studies reporting changes in uterine position of up to 48 cm (mean 7 cm) in patients with cervical and endometrial cancer [21]. Movement was graded semi-quantitatively as follows: no movement; up to 5 mm; and more than 5 mm. This gentle displacement equated to approximately 5 N tension when this was applied with haemostats attached to a spring gauge. A further series of measurements was then done on 6 male and 6 female cadavers to quantify the amount of endopelvic movement produced by 5 N tension. After initial gentle pulling to determine the plane in which most traction and/or movement of the endopelvic fascia was seen, all tension on the structure was released and the inherent elasticity of the endopelvic fascia allowed it to retract back to its original position. A partially-opened 32 mm round stainless steel paperclip was placed on the surface of the endopelvic fascia near to the organ to be pulled, and the straight edge of the paperclip was aligned at right angles to a small perspex millimetre ruler which was held as close as possible to the paperclip. Gentle tension was applied to the structure through haemostat forceps clamped superficially over 2-3 mm of the structure of interest. A spring hook tension gauge was attached to one finger ring of the haemostat and by gently pulling on this gauge tension was gradually increased to 5 N and the movement of the paper clip was recorded. At no point was the displacement sufficient to produce disruption of the connective tissue elements surrounding the viscera. The mean of three measurements per visceral structure in each cadaver was used for subsequent analysis. Reliability of the measurement methods was assessed for tension applied to the rectosigmoid junction (2 male and 2 female cadavers) and the prostate (4 male cadavers). In this case, 10 measurements for each structure were made by the same observer by moving sequentially from cadaver to cadaver.

## Results

### Reliability of measurements

Maximum and minimum measurements of endopelvic fascial movement in 4 male cadavers for tension applied to the rectosigmoid junction, where marked movement was seen, varied from 12% to 27% of the mean, and the standard deviations for different cadavers varied from 7% to 17% of the mean (Table 1). In contrast, maximum and minimum measurements for tension applied to the prostate in the same cadavers, where little or no movement was seen, had a much larger range, varying from 41% to 74% of the mean with similarly large standard deviations (41% to 50% of the mean).

**Table 1:** Movement (mm) of the endopelvic fascia on pulling the rectosigmoid junction (RSJ) or the prostate gland

Trial	RSJ 1	RSJ 2	RSJ 3	RSJ 4	Prostate 1	Prostate 2	Prostate 3	Prostate 4
1	22	20	11	28	3	3	2	1
2	29	20	10	28	4	1	1	1
3	30	18	15	26	2	4	2	3
4	19	22	13	29	1	2	3	2
5	20	20	16	25	1	3	1	1
6	22	18	14	26	2	5	1	4
7	21	21	17	25	3	4	1	4
8	25	20	12	26	2	3	2	3
9	29	18	12	22	3	2	3	4
10	29	19	14	24	2	2	1	2
*n*	*10*	*10*	*10*	*10*	*10*	*10*	*10*	*10*
*mean*	*24.60*	*19.60*	*13.40*	*25.90*	*2.30*	*2.90*	*1.70*	*2.50*
*max*	*30*	*22*	*17*	*29*	*4*	*5*	*3*	*4*
*min*	*19*	*18*	*10*	*22*	*1*	*1*	*1*	*1*
*Sdev*	*4.30*	*1.35*	*2.22*	*2.08*	*0.95*	*1.20*	*0.82*	*1.27*

### Movement of endopelvic fascia

With adequate lighting, movement of the endopelvic fascia in intact cadavers where the abdomen only had been opened could be seen for all the structures studied. The restricted access provided by such specimens, however, made reliable measurements impossible. All quantitative studies were therefore made on cadavers where the pelvis had been sectioned in the sagittal plane.

Semi-quantitative observations of 12 male and 6 female cadavers showed that most movement of the endopelvic fascia was produced by gently pulling on the rectosigmoid junction or the uterus near the broad ligament (Table 2). The plexus did not cross any vertebral joints and there was no visible movement of the endopelvic fascia when the hypogastric nerve contribution to the plexus was pulled.

**Table 2:** Most common band of movement of the endopelvic fascia observed after pulling on pelvic viscera

Structure	Relationship to plexus	No movement	Up to 5 mm	More than 5 mm
Rectosigmoid junction	Anterolateral			√
Uterine tube/broad ligament	Anterolateral			√
Ovary/mesovarium	Anterior		√	
Seminal vesicles	Anterior		√	
Prostate	Anteromedial		√	
Uterus (near cervix at the rectouterine pouch)	Anterosuperior		√	
Sigmoid colon/sigmoid mesocolon	Lateral	√		
Hypogastric nerve	Superior	√		
Superior rectal artery	Superolateral	√		
Trigone of bladder	Anterosuperior	√		
Uterus (fundus)	Anterosuperior	√		
Rectum (superior third)	Inferiomedial	√		

**Figure 1:**
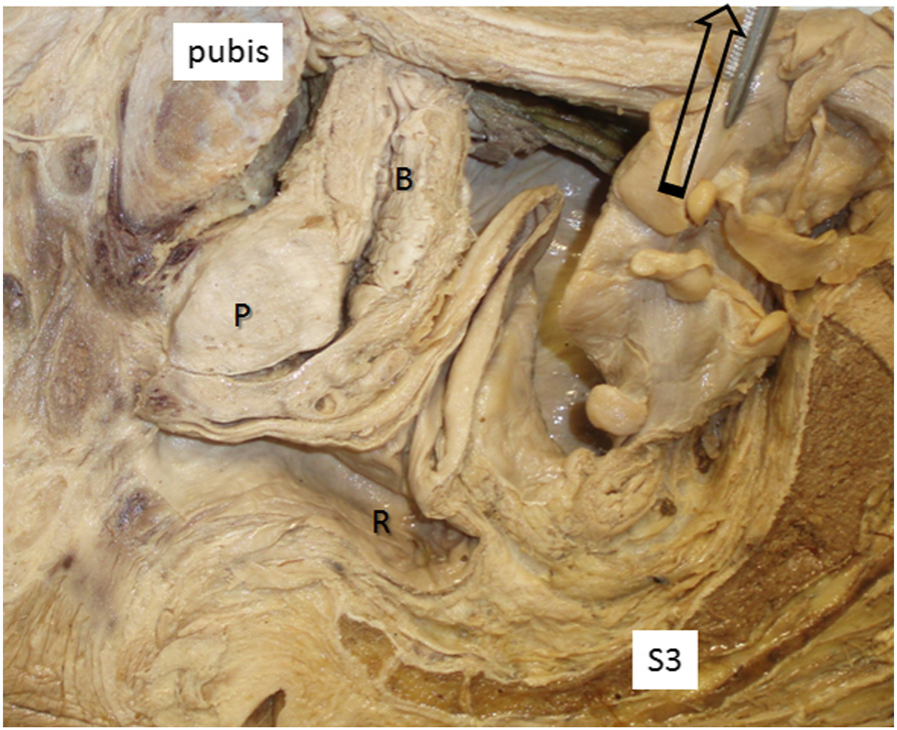
**Anterior reflection of the rectosigmoid junction in the male by 5 N traction of the sigmoid colon.** This procedure caused tension and movement of the endopelvic fascia overlying the inferior hypogastric plexus (*) which lies anterior to the 3^rd^ sacral vertebra (S3). Key to Figures 1, 2, 3, 4. B: bladder; P: prostate; V: vagina; R; rectum.

**Figure 2:**
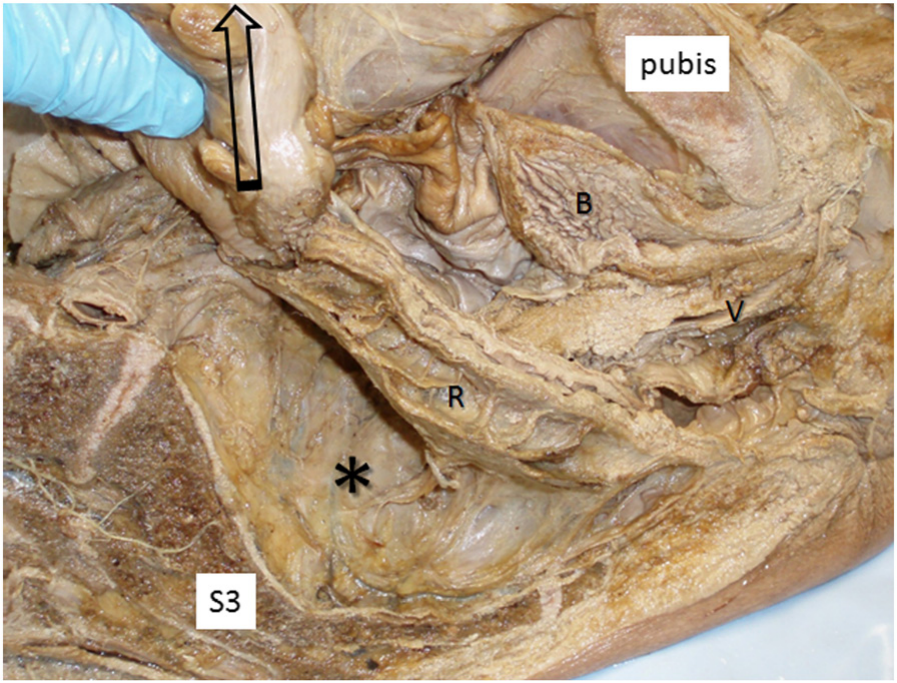
**Anterior reflection of the rectosigmoid junction in the female.** The position of the inferior hypogastric plexus in the endopelvic fascia is indicated (*).

**Figure 3:**
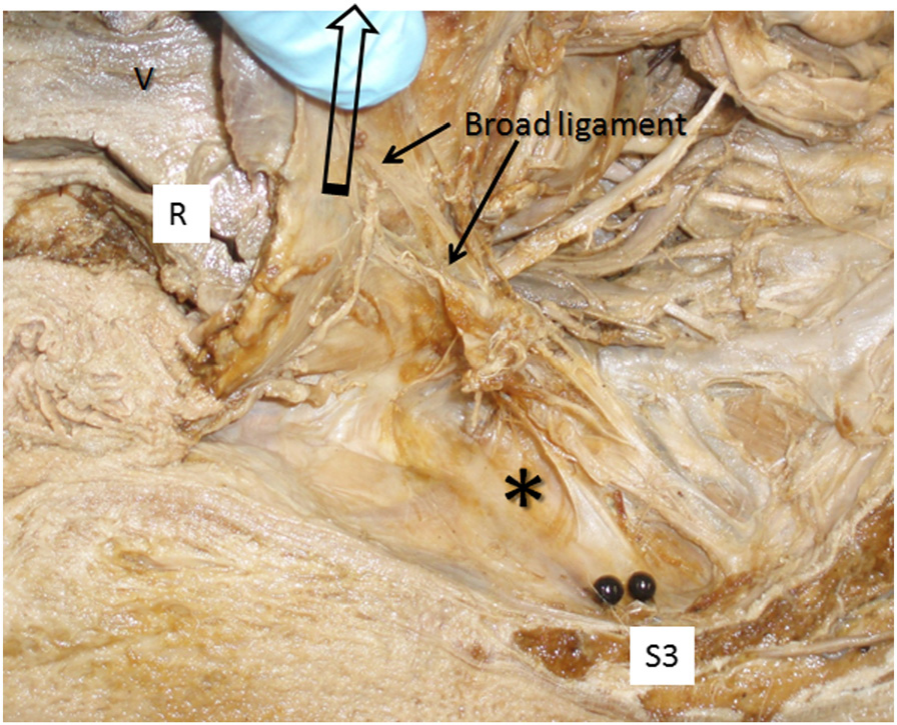
**Anterior reflection of the broad ligament of the uterus containing the uterine tube.** The position of the inferior hypogastric plexus in the endopelvic fascia is indicated (*).

**Figure 4:**
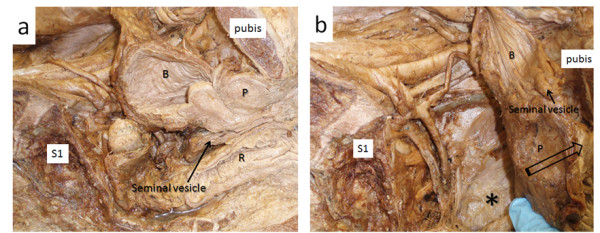
**a. Undisturbed view of the right seminal vesicle extending posterolaterally from the prostate gland. b.** Manual anterior reflection of the seminal vesicle shown in Figure 4a. The position of the inferior hypogastric plexus in the endopelvic fascia is indicated (*).

Table 3 summarises mean movement of the endopelvic fascia containing the inferior hypogastric plexus caused by up to 5 N tension on different visceral structures. In some specimens structures were either absent or had been damaged by the pelvic hemisection procedure and they were not included. Most (≫5 mm) movement of the endopelvic fascia overlying the inferior hypogastric plexus was measured after applying tension to the rectosigmoid junction (Figures 1 &2) and the broad ligament at the level of the infundibulum of the uterine tube (Figure 3). Both these structures were sited anterolateral to the plexus. Moderate (up to 5 mm) movement was measured after applying tension to structures that were located anterior to the plexus, such as the ovary via its mesovarium, the uterus near the cervix at the rectouterine pouch, the prostate gland and the seminal vesicles (Figure 4). Structures for which tension up to 5 N had no visible effect on the endopelvic fascia were located anterosuperiorly (uterine fundus, trigone of bladder), superolaterally (superior rectal artery) laterally (sigmoid colon via its mesocolon), inferiomedially (proximal third of rectum) and superiorly (hypogastric nerve).

**Table 3:** Mean movement (mm) of endopelvic fascia on pulling pelvic viscera

Male/female	Rectosigmoid junction	Uterine tube/ broad ligament	Ovary/ mesovarium	Seminal vesicle	Prostate	Uterus
F	20	35	5			3
F	18	18	3			3
F	27	20	3			2
F	14					3
F	8					4
F	17	15	4			2
M	11			3	1	
M	9			5	3	
M	8			5	4	
M	13			2	1	
M	22			2	1	
M	12			4	1	
*n*	*12*	*4*	*4*	*8*	*8*	*6*
*mean*	*14.97*	*22.00*	*3.75*	*3.38*	*2.00*	*2.83*
*max*	*27.33*	*35*	*5*	*5*	*4*	*4*
*min*	*8.33*	*15*	*3*	*2*	*1*	*2*
*Sdev*	*5.93*	*8.94*	*0.96*	*1.19*	*1.07*	*0.75*

## Discussion

This study shows that most movement of the region of the endopelvic fascia containing the pelvic plexus is produced by applying tension to visceral structures located anterolateral to the plexus. These anterolateral structures include the rectosigmoid junction and broad ligament of the uterus. Similar movement might be expected of the more pliable fascia of the living, possibly as a consequence of menstruation, pregnancy or chronic constipation.

### Reliability of data

The quantitative data presented for groups of 4 or 12 cadavers supports the semi quantitative qualitative observations made on 18 cadavers where broad categories of movement of the endopelvic fascia containing the inferior hypogastric plexus were documented and this is in line with qualitative observations made on the whole sample of 28 cadavers. In addition, the variability of values obtained when the measured movement was over 5 mm gave a relative standard error of the mean (RSE) of 3.4-8.7%, indicating good reproducibility. It would therefore seem reasonable to conclude that most endopelvic fascial movement is produced by tension of rectosigmoid junction and broad ligament. For movement in the 1-5 mm range, however, the RSE was 20.7-25.4, indicating that these measurements are unreliable with the degree of precision possible with the present method.

The process of embalming will result in stiffening of connective tissue elements, presumably through the formation of methylene bridges between the aldehyde group of formalin in the fixative and the amino groups of proteins such as collagen [22]. Some coagulation of proteins would also be expected due to the presence of methanol and phenol in the fixative. This hardening effect was clearly present in muscles and skin of the specimens examined here. The loose irregular connective tissue of the endopelvic fascia, however, was much less affected and retained its elasticity such that the gentle pulling done in this study was followed by the return of the facia to its original position. This could be taken to indicate that the endopelvic fascia in the living will stretch even further than that in the cadaver, making it more likely that the nerve fibres it contains will be stretched. The predominance of collagen over elastin in the sheaths of peripheral nerves [23] could render them vulnerable to mechanical damage by such stretch. It is emphasised, however, that the degree to which this reflects the mechanical properties of endopelvic fascia (i) in the living and (ii) in the intact- as opposed to the hemisectioned- pelvis remains to be determined. This could be done directly at surgery or through the use of soft tissue imaging in association with endoscopic-assisted rectosigmoid movement. It is known, for example, that several centimetres of large bowel movement are possible before pain limits the advancement of a rigid sigmoidoscope, and that the positions of both the colon and uterus vary considerably in life [24-26].

### Tension of inferior hypogastric nerve fibres

The endopelvic fascia contains the inferior hypogastric plexus and its fine nerve branches [17,27]. Since this fascia also blends with the coverings of many pelvic organs, their movement will indirectly exert a pull on the plexus. Structural and functional sequelae of adverse neural tension are well known [13,14,28,29]. It has also been suggested, on the basis of anatomical studies of three embalmed female cadavers, that damage to the inferior hypogastric plexus during vaginal delivery or sustained constipation may be associated with a variety of obstetric and gynaecological syndromes [30]. Notwithstanding these observations, it seems unlikely that normal visceral movement would result in sufficient tension to adversely affect inferior hypogastric nerve fibre function. Normal nerve movement in association with local inflammation, however, may be sufficient to affect nerve function, as it has been shown that local inflammation of hind limb nerves in rats causes their axons to exhibit abnormally high mechanosensitivity when subjected to stretch within normal ranges [15].

The inferior hypogastric plexus is consistently found embedded in the endopelvic fascia one-third of the way along- and deep to- a line drawn from the presumptive recto-sigmoid junction at the level of the third sacral vertebra and the palpable posterior superior surface of the pubic symphysis [17]. In this position, the plexus did not cross any joints and was related to the sacrum only indirectly, as the levator ani muscle always intervened. It is unlikely, therefore that any slight movement at the sacroiliac joint would exert any significant traction on the plexus. It is possible that the greater range of movement at the lower lumbar and lumbosacral joints might exert some tension on the plexus via the endopelvic fascia or the hypogastric nerve whose T11-L2 nerve roots provide a sympathetic contribution to the hypogastric plexus [10], but this could not be demonstrated in the embalmed cadaver where tissue and joint stiffness is markedly increased.

A relationship between somatic and autonomic nervous system function has featured in several manual therapy theories to explain the effects of spinal manipulation on visceral function [9,31]. Work on experimental animals has shown that somatovisceral responses are consistently evoked through stimulation of C-fibres by noiciception, and through hyperstimulation of proprioceptors through movement of joints beyond their normal physiological range, as is common during joint manipulation [32]. Evidence of altered sympathetic activity has also been reported in humans after mobilisation of the elbow in lateral epicondylagia [33]. These somatovisceral responses have generally been explained by viscerosomatic convergence of afferent input in laminae I to V of the dorsal horn [34]. Many theories on the mechanism of manual therapy now include distant autonomic effects [35]. It has been reported for cats and rats that nociceptive stimulation of viscera leads to expansion and lowered excitation thresholds of somatic afferents converging in the dorsal horn and vice versa [34,36,37]. We have found tyrosine hydroxylase- positive (sympathetic) nerve fibres in the inferior hypogastric plexus in humans [17], that are presumably derived from the hypogastric nerve (T11-L2). Sympathetic afferents have long been presumed to convey pain sensations from the intervertebral discs via the L2 nerve root [38] and it is well-established that low back pain accompanies many pelvic visceral pathologies [39]. These studies provide evidence for an interaction between autonomic and somatic nerve activity. Observations from this study suggest that the visceral movement, particularly that of the uterus or rectosigmoid junction, might also affect this interaction, possibly as a consequence of menstruation, pregnancy or constipation. Enlargement of the colon diameter from approximately 3 cm to ≥ 5 cm is generally taken to be diagnostic of colonic obstruction of which constipation is one cause [40], while enlargement beyond 10 cm is typically seen in aganglionic megacolon, with various degrees of enlargement in between for other congenital conditions including vovlvulus and sigmoidocoele [41]. Similarly, the uterus elongates by approximately 3 cm during the menstrual cycle [42]and the uterine fundus near term reaches the xiphisternum from a non-gravid retropubic position [43], a change which involves a massive 4–5 times increase in length. Other pelvic floor disorders such as pelvic descent have been attributed to paradoxical contraction of muscles as a result of perturbed feedback, although the precise neural mechanism remains unclear [44-48]. In particular, it is possible that the small nerve branches that ramify in the endopelvic fascia are affected differently by stretch compared to large nerves. For example the large pudendal nerve is variously described as a casualty [44] or an epiphenomenon [47] of an unknown change in neuromuscular function that affects the process of defaecation. If the primary nerve damage, however, is to small nerve fibres which travel via several large nerves to influence lumbosacral spinal reflexes then the poor correlation of large nerve damage might be explained. A possible mechanism underlying a change in excitability of damaged small nerve fibres is increased sensitivity to mechanical stimuli due to the accumulation of mechanosensitive ion channels at the sites of locally impaired axonal transport [49]. Whether accompanying local inflammation is necessary or sufficient to affect the activity of nerve fibres within the fascia surrounding these viscera is presently unknown, but could possibly be addressed by correlative studies of the somatovisceral features of normal visceral movement (e.g. uterine changes during menstruation) with or without associated local inflammation (e.g. pelvic inflammatory disease). Marked displacement of viscera with inevitable endopelvic fascial stretch is a feature of most abdominopelvic operative procedures [50] and methods have already been developed in the living to investigate large bowel function by combining measurements of pressure and muscle and nerve electrical activity with ultrasound and magnetic imaging [51]. It should be possible, therefore, to adapt these approaches to study pelvic organs during elective surgery so that smooth muscle and visceral nerve activity can be compared under undisturbed conditions and conditions where these organs are displaced, either manually by the surgeon, using the data provided in this study, or paraphysiologically through infusion of fluid into the lumina of hollow viscera.

## Conclusions

This study shows that the endopelvic fascia containing the fine branches of the inferior hypogastric plexus can be displaced up to 35 mm by mild (5 N) traction of pelvic viscera, in particular the distal colon and uterus which are well-known for changing size and position. This could result in autonomic nerve tension. This is important as it provides a structural basis that can be used in support of earlier somatovisceral reflex theories invoked to explain the involvement of visceral pathology in the generation of somatic pain and the amelioration of the former by manual therapy. Thus, there is evidence from animal studies that mild nerve tension in the presence of local inflammation results in altered nerve function [15] and that there is convergence of somatic and visceral neural inputs at the spinal cord level [34]. Moreover, there are limited reports that visceral referred pain can be reduced by applying anaesthesia to the somatic site of referral [52,53]. This, together with the results of the present study, provides (i) a mechanism by which visceral movement results in pain that is referred to somatic structures and (ii) a pathway by which stimulation of somatic nerves by manual therapy might reduce the perceived intensity of such visceral referred pain. This enables clearer hypotheses to be generated for testing in the living.

## Competing interests

The author declares that they have no competing interests.

## Authors’ contributions

The author conceived the study, carried out the research and wrote the manuscript.
